# Toward Greener Rubber: Impact of Resin Type and Amount on Curing, Network Structure, and Viscoelastic Properties in SBR compounds

**DOI:** 10.1021/acspolymersau.6c00018

**Published:** 2026-03-19

**Authors:** Michele Pierigé, Francesca Nardelli, Mattia Cettolin, Andrea Causa, Luca Giannini, Francesca Martini, Lucia Calucci, Marco Geppi

**Affiliations:** † Dipartimento di Chimica e Chimica Industriale, 9310 Università di Pisa, Via G. Moruzzi 13, 56124 Pisa, Italy; ‡ Istituto di Chimica dei Composti OrganoMetallici, 9327 Consiglio Nazionale delle Ricerche, Via G. Moruzzi 1, 56124 Pisa, Italy; § Centro per l’Integrazione della Strumentazione Scientifica dell’Università di Pisa (CISUP), Lungarno Pacinotti 43/44, 56126 Pisa, Italy; ∥ 433522 Pirelli Tyre SpA, Viale Sarca 222, 20126 Milano, Italy

**Keywords:** tackifying resins, bio-based additives, glass transition, polymer−resin miscibility, cross-linking, marching effect, solid-state NMR

## Abstract

In the pursuit of more sustainable tire technologies, the replacement of petroleum-derived additives with bio-based alternatives has become a key research focus. Tackifying resins, which contribute significantly to tread performance and viscoelastic behavior, are among the additives under evaluation for substitution. In this study, several complementary techniques were applied to styrene–butadiene rubber (SBR) compounds, containing either a natural rosin-based resin (Dertoline) or a petroleum-derived resin (Kristalex) at concentrations ranging from 15 to 45 phr, to investigate the effect of resin on the curing process and on compound properties before and after curing. Solid-state NMR spectroscopy and differential scanning calorimetry revealed intimate mixing between polymer and resin at all concentrations and a slowdown in dynamics with increasing resin content that was more pronounced for Kristalex. Dertoline appeared to hinder effective interactions between carbon black, used as a filler, and SBR, leading to a strong suppression of bound rubber, as evidenced by swelling experiments. Vulcanization kinetics, monitored by moving die rheometer experiments, was affected by the two resins in different manners: Kristalex slowed down curing, while Dertoline accelerated it. Moreover, a marching phenomenon was observed at long curing time for high Dertoline concentrations. Cross-link density, determined by equilibrium swelling, decreased with increasing resin loading. Final mechanical and viscoelastic properties of vulcanized compounds, evaluated by dynamic mechanical analysis and tensile tests, were influenced by both resin type and concentration, showing mechanical plasticization effects and changes in both the elastic and anelastic response. Importantly, the two resins appeared to influence wet grip and rolling resistance differently, highlighting their distinct impact on key performance parameters. These results support the tailored use of bio-based tackifiers in sustainable SBR formulations.

## Graphical abstract

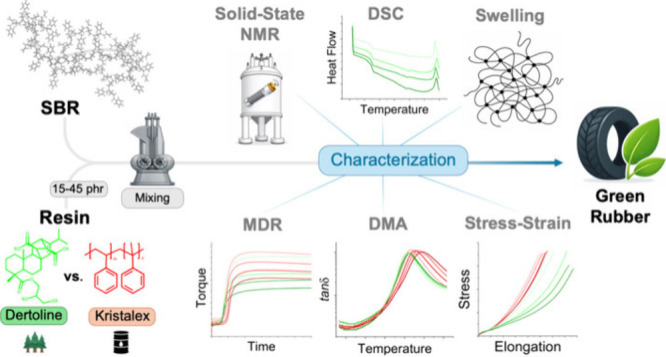

## 1 Introduction

The increasing demand for sustainable solutions in the manufacturing sector is driving research toward innovative materials and processes that align with the goals of the energy transition. In this context, rubber technology in the automotive industry is undergoing a significant transformation, aimed not only at reducing the environmental impact of tire production, but also at enhancing energy efficiency throughout the entire product lifecycle.^1–6^ This transformation encompasses multiple stages of the production chain, including raw material selection, manufacturing processes, tread design, as well as the treatment and recycling of end-of-life tires.

Tire rubber compounds are complex mixtures with the primary constituents being the elastomeric matrix, vulcanization agents, tackifiers, and plasticizers. Currently, most of these materials derive from fossil resources. In line with global efforts toward carbon neutrality, many major tire manufacturers have committed to using 100% sustainably sourced materials in their products by 2050.^5^ Sustainable materials are generally defined as those that are either bio-based or recycled, with bio-based referring to substances wholly or partially derived from renewable biological sources.

Significant research efforts are underway to develop and integrate alternative materials into rubber compounds.^2–6^ Natural rubber from various plant sources,^2,7–9^ along with synthetic rubber derived from bio-based monomers and biomass,^10–14^ has been explored as an alternative to conventional fossil-based synthetic polymers. In addition to the rubber matrix, replacing traditional additives with more sustainable alternatives is also essential. Several studies have focused on incorporating environmentally friendly reinforcing fillers of natural origin^15,16^ or recycled from waste materials.^17^ Eco-friendly process oils, such as mild extraction solvate and treated distillate aromatic extract (TDAE), which contain low levels of polycyclic aromatic compounds and polyaromatic hydrocarbons, as well as vegetable oils, have been tested as substitutes for conventional aromatic and naphthenic process oils.^5,18,19^

A key challenge is to ensure that the use of sustainable materials does not compromise tire performance, typically evaluated through the so-called “magic triangle”,^20–22^ which involves the simultaneous optimization of rolling resistance, wet grip, and tread life. These properties result from a complex interplay of factors and interactions occurring at both the molecular and supramolecular levels. Therefore, it is crucial to investigate the effects of adding alternative materials not only on the mechanical and viscoelastic behavior of rubber compounds, but also on the structural and dynamic properties of the polymer network, which are at the origin of the final characteristics.

Tackifying resins are essential components in rubber compounds, playing a key role in enhancing the performance of tire treads.^22,23^ They are typically low molecular weight solids with a high softening point. Optimized tack is crucial in tread formulations to achieve better grip, wear resistance, and long-term durability. Tackifiers influence the viscoelastic behavior of rubber compounds, improving filler dispersion, processability, and internal adhesion between different components, in a way that depends on the chemical structure and loading of the resin itself.^24–26^ Recently, there has been increasing interest in bio-based tackifiers derived from renewable resources, offering a more sustainable alternative to traditional petroleum-based products.^27^ In this context, plant-based resins, such as rosin resins, terpene-based resins, and biophenolic resins, have been tested as multifunctional additives in rubber compounds, and their effects on curing behavior, rheological properties, mechanical strength, and adhesion have been evaluated.^28–33^

In a previous study by some of the authors,^33^ the natural rosin-based resin Dertoline was investigated as a sustainable alternative to conventional petroleum-derived tackifiers in styrene–butadiene rubber (SBR) compounds filled with carbon black (CB). The research focused on samples containing 15 phr resin and compared the effects of the natural resin with those of a petroleum-derived aromatic resin (Kristalex) and a phenolic resin (SMD). The influence of these additives on the curing kinetics, cross-link density, and polymer–resin compatibility was assessed using techniques such as moving die rheometry (MDR), swelling experiments, differential scanning calorimetry (DSC), dynamic mechanical analysis (DMA), and solid-state nuclear magnetic resonance (SSNMR). The results showed that the natural resin had a performance comparable to those of the synthetic ones, supporting its potential for industrial applications.

This study expands upon the previous work^33^ by investigating SBR-based compounds filled with CB, in which the resin content was systematically varied from 15 to 45 phr, while maintaining the same base formulation as the earlier study. Samples without CB were also studied to decouple resin-related effects from those due to filler–rubber interaction. The effect of increased resin loading on the material’s microscopic and macroscopic properties was explored by considering two types of resins: the natural rosin-based resin Dertoline and the petroleum-derived resin Kristalex, whose structures are shown in Figure 1. Unlike the previous work,^33^ where mixing was performed using a Banbury mixer, the compounds were processed with a Brabender mixer. A comprehensive set of experimental techniques, including SSNMR, DSC, MDR, DMA, stress–strain and swelling tests, was employed to investigate the effect of resin type and content on properties of uncured and cured compounds, as well as on the curing process. Investigations on uncured compounds focused on phase behavior, dynamic properties, sample homogeneity, and resin–CB interactions. SSNMR spectroscopy, combining high-resolution ^13^C selective experiments and time-domain ^1^H relaxation measurements, was used to study phase structure and molecular dynamics. DSC assessed thermal behavior and polymer–resin compatibility, while swelling experiments provided insights into filler-resin interactions and bound rubber formation. The effect of resin addition on vulcanization kinetics was evaluated by MDR experiments at 443 K, monitoring the torque evolution during cross-linking. After curing, the cross-link density of the vulcanized compounds was assessed by equilibrium swelling, while viscoelastic and mechanical properties were characterized through temperature-sweep DMA and stress–strain tests. This comprehensive investigation offers valuable insights into mixing efficiency, polymer–resin compatibility, curing behavior, and CB–resin interactions, deepening the understanding of how bio-based and synthetic resins affect SBR formulations and the complex interplay among their components.

**Figure 1:**
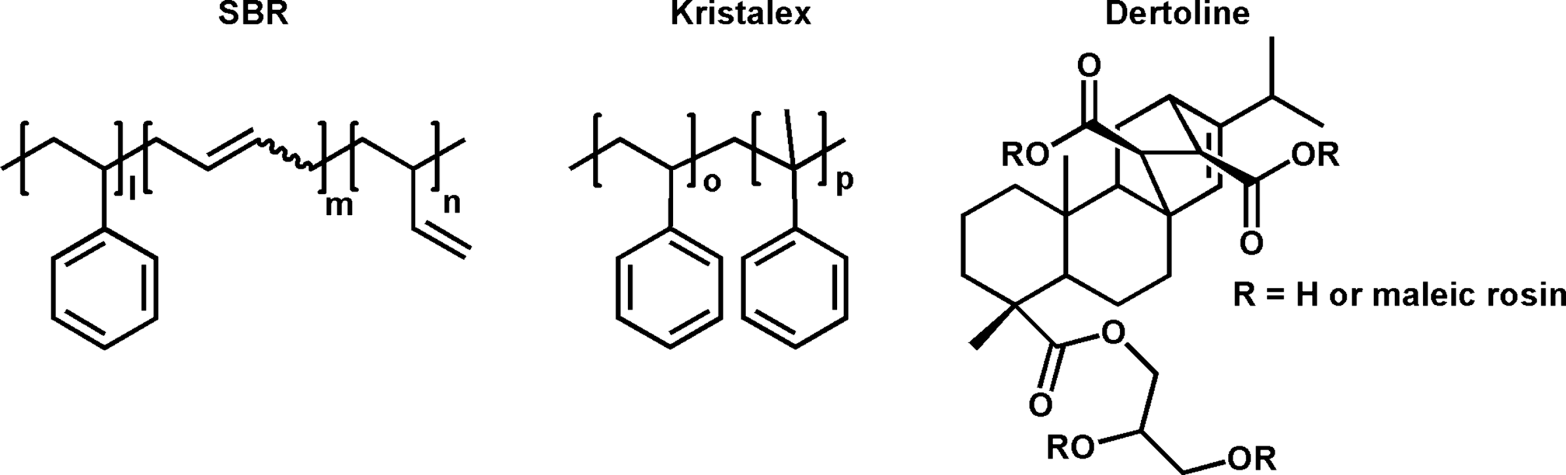
Chemical structures of styrene–butadiene rubber (SBR) and Kristalex and Dertoline resins.

## 2 Experimental Section

### 2.1 Materials

The compounds object of this study were produced at Pirelli Tyre SpA (Milan, Italy). Each compound was examined in both cured and uncured forms.

The polymeric matrix consisted of solution SBR (S-SBR; *M*_n_ = 530 000 g/mol; *M*_w_ = 750 000 g/mol; styrene content = 39.5 wt %; vinyl content in the diene fraction = 38.5 wt %) extended with TDAE oil. Formulations containing different types and amounts of tackifying resins were investigated. The tackifying resins used were Kristalex 5140 (Synthomer, Essex, U.K., *M*_n_ = 1690 g/mol, *M*_w_ = 4750 g/mol) and Dertoline MG (DRT, Dax, France, *M*_n_ = 2000 g/mol, *M*_w_ = 4200 g/mol), which are hereafter termed Kristalex and Dertoline, respectively. All compounds contained a vulcanization package consisting of sulfur, zinc oxide, and *N*-cyclohexyl-2-benzothiazole sulfenamide (CBS) as accelerators, and stearic acid as an activator. Carbon black (CB, N100 series) was used as reinforcing filler. The formulations and the corresponding sample codes are reported in Table 1.

**Table 1:** Formulations of the Investigated Compounds

	content (phr)^a^								
ingredient	REF	K15	K25	K35	K45	D15	D25	D35	D45
SBR	100	100	100	100	100	100	100	100	100
TDAE	37.5	37.5	37.5	37.5	37.5	37.5	37.5	37.5	37.5
CB	45	45	45	45	45	45	45	45	45
zinc oxide	3.5	3.5	3.5	3.5	3.5	3.5	3.5	3.5	3.5
stearic acid	2	2	2	2	2	2	2	2	2
Kristalex	–	15	25	35	45	–	–	–	–
Dertoline	–	–	–	–	–	15	25	35	45
CBS	4	4	4	4	4	4	4	4	4
sulfur	2	2	2	2	2	2	2	2	2
total	194	209	219	229	239	209	219	229	239

a phr = part per hundred rubber.

The compounds were prepared using a two-stage mixing process with a Brabender internal mixer featuring a 55 mL mixing chamber. The first stage was conducted at 60 rpm. The TDAE-extended SBR was mixed for 30 s. Then, a partial amount of resin, a variable quantity of filler (25–40 phr) depending on the resin content, zinc oxide, and stearic acid were added and incorporated into the rubber matrix for 30 s. After that, an additional portion of resin and the remaining filler were introduced, followed by further mixing for 1.5 min. Finally, the remaining resin was added, and the system was blended for 3.5 min. The second stage was carried out the following day at 45 rpm. The compounds were mixed for 30 s before CBS and sulfur were introduced. Additional mixing continued for 2 min, reaching a dumping temperature of 373 K to prevent premature vulcanization. Curing was performed at 443 K for 10 min on samples in the form of 10 × 10 cm^2^ sheets with a thickness of 1 mm.

Samples with the same composition and vulcanized in the same conditions but without CB are indicated using the same name and the suffix “_CF”, which stands for carbon-black-free.

For vulcanized samples, the letter “v” is added as a prefix to the code used for the uncured compounds.

### 2.2 SSNMR Experiments

^13^C experiments and measurements of ^1^H spin–lattice relaxation times (*T*_1_) and spin–lattice relaxation times in the rotating frame (*T*_1ρ_) were performed using a Bruker Avance Neo 500 spectrometer, operating at ^1^H and ^13^C Larmor frequencies of 500.13 and 125.76 MHz, respectively. A double-resonance 4 mm cross-polarization (CP)–magic angle spinning (MAS) probe was employed. The 90° pulse durations for ^1^H and ^13^C were 4.3 and 3.76 μs, respectively.

High-resolution ^13^C spectra were acquired at a MAS frequency of 5 kHz. Both ^13^C CP/MAS and delayed CP/MAS experiments were performed using the CP contact times (ct) specified in the text, with a recycle delay (rd) of 4 s. A total of 800 scans were accumulated for CP/MAS and 2000 scans for delayed CP/MAS experiments. In the delayed CP/MAS experiments, a delay of 100 μs was introduced between the 90° excitation pulse and the CP block. ^13^C DE/MAS spectra were recorded with an rd of 2 s, accumulating approximately 2000 scans.

^1^H *T*_1_ and *T*_1ρ_ relaxation times were measured under static conditions in the 303–343 K temperature range. ^1^H *T*_1_ values were measured using an inversion recovery pulse sequence with variable relaxation delay values ranging from 0.001 to 5 s. For each delay, 16 scans were accumulated using a recycle delay of 5 s. ^1^H *T*_1ρ_ relaxation times were measured using a variable spin-lock time pulse sequence by applying a 90° pulse followed by a spin-lock pulse with variable duration in the 0.05–18 ms interval. The spin-lock field (ω_1_/2π) was 46 kHz. The recycle delay was 4 s, and four scans were accumulated for each experiment.

^1^H on-resonance free induction decays (FIDs) and Carr–Purcell–Meiboom–Gill (CPMG) curves were recorded at 303 K using a Niumag permanent magnet working at the ^1^H Larmor frequency of 20.8 MHz, interfaced with a Stelar PC-NMR console. A single-channel static 5 mm probe was employed. The ^1^H 90° pulse duration was 3.3 μs. ^1^H FIDs were acquired using the mixed magic sandwich echo (MSE) pulse sequence.^34^ The total echo duration was set to 6­(4τ_φ_ + 2τ_90_), with τ_φ_ = 1.5 μs and τ_90_ = 3.3 μs. The recycle delay was 0.5 s, and 200 scans were accumulated. ^1^H CPMG curves were acquired using the alternating-phase CPMG pulse sequence^35^ to measure transverse magnetization decay without spin-locking artifacts. For each curve, one data point was collected every four Hahn-echo blocks using an echo delay of 15 μs, for a total of 200 points. A relaxation delay of 0.5 s was used, and 128 scans were accumulated. To accurately determine the *T*_2_ of long-decaying components in the FID, the analysis was performed on a “pseudo” FID constructed by combining MSE FID data for times shorter than 150 μs with CPMG data for times exceeding 150 μs. The resulting curves were analyzed using a discrete approach based on a nonlinear least-squares fitting procedure implemented in the Mathematica environment.^36^

To aid the interpretation of relaxation data in terms of sample mobility, particularly in the presence of multiexponential relaxation behaviors or distinct components with different intrinsic relaxation times, a medium relaxation time (*T*_km_, with k = 1, 2, 1ρ) was calculated as the inverse of the population weighted rate average (PWRA­(*T*_k_)):

$$\mathrm{PWRA}\left(T_{k}\right)=\underset{i}{\sum }\frac{f_{i}}{T_{\mathrm{ki}}}$$ 1

where *T*_ki_ is the relaxation time of the ith component and *f*_i_ is the corresponding proton fraction.

### 2.3 Determination of Bound Rubber Percentage

The percentage of bound rubber (BdR) was determined for uncured compounds as follows. Approximately 0.5 g of compound was weighed and placed in a Pirex gooch crucible (porosity grade 1). The sample was immersed in toluene for 72 h, with the toluene being refreshed every 24 h. The residual undissolved sample was then dried and weighed. The BdR was calculated using the following equation:^37^

2 $$\mathrm{BdR}=\frac{W_{d}-W_{\mathrm{CB}}}{W_{\mathrm{SBR}}}\times 100\%$$

where *W*_d_ is the weight of the dried residual sample after swelling, containing both filler and bound rubber, and *W*_CB_ and *W*_SBR_ are the initial weights of the filler and the rubber in the compound, respectively.

### 2.4 DSC Experiments

DSC experiments were carried out using a Mettler Toledo 823e+ instrument (Mettler-Toledo S.p.A., Milan, Italy). Thermal scans were conducted over the 183–473 K temperature range, with a cooling and heating rate of 10 K/min. The glass transition temperature (^DSC^ *T*_g_) was identified as the temperature corresponding to the inflection point of the DSC curve during the heating cycle. For REF and pure resins, the change in heat capacity at the glass transition (Δ*C*_p_) was determined from the DSC heating curve as the difference in baseline heat flow before and after *T*_g_, measured at the inflection point.

### 2.5 MDR Experiments

The curing behavior of the compounds was evaluated using a moving die rheometer (RPA 2000, Alpha Technologies, London, U.K.). The experiments were performed with an oscillation angle of ±1° under a pressure of 4.3 bar at a temperature of 443 ± 1 K, maintained for 30 min. The following parameters were recorded for each test: minimum torque (*M*_L_), maximum torque (*M*_H_), optimum cure time (*t*_c90_), and scorch time (*t*_s2_). To evaluate the kinetic behavior of the different compounds, the cure rate index (*CRI*)^38^ was calculated according to the following equation:

3 $$\mathrm{CRI}=\frac{100}{t_{c90}-t_{s2}}$$

The cure time (*CT*) was calculated as the intersection point between the two tangent lines A and B to the MDR derivative curve (see Figure S1) plus an additional 1 min.^39^

### 2.6 Equilibrium Swelling Experiments

Equilibrium swelling experiments were carried out on the vulcanized samples in duplicate to determine the total cross-link density, *M*_c_^–1^, where *M*_c_ represents the average molar mass between two cross-links. Following the Flory–Rehner method, each sample was weighed: (i) initially, (ii) after 72 h of immersion in toluene in the dark, and (iii) after drying overnight in an oven at 343 K under vacuum. Based on these three measurements, the *M*_c_^–1^ values were calculated using the Flory–Rehner equation.^40,41^

### 2.7 Stress–Strain Experiments

Tensile strength tests were carried out on dumbbell-shaped specimens in triplicate according to ISO 37:2017 standards,^42^ using an Instron 5800 apparatus at 298 K, with a crosshead speed of 500 mm/min. The measured parameters, determined as the average of the three tests, included the moduli at 10%, 20%, 50%, 100%, 200%, and 300% elongation (*M*_10_, *M*_20_, *M*_50_, *M*_100_, *M*_200_, and *M*_300_), tensile strength at break (*TS*_b_) and elongation at break (*E*_b_).

### 2.8 DMA Experiments

Temperature sweep measurements were carried out on cured samples using a Metravib DMA + 1000 (Acoem) instrument by a tensile stress mode. Test specimens were prepared by cutting 30 mm × 10 mm rectangular strips from 1 mm thick compound sheets. The temperature dependence of the complex elastic modulus was measured by oscillatory tensile deformation at a frequency of 1 Hz with 0.1% strain deformation, and at the heating rate of 2 K/min.

Then, *tan*δ was calculated as the ratio of the loss modulus (*E*″) to the storage modulus (*E*′). The glass transition temperature (^DMA^ *T*_g_) was taken as the temperature at the maximum of the *tan*δ peak.

## 3 Results and Discussion

### 3.1 Phase Properties and Dynamics by SSNMR

An SSNMR investigation was carried out to examine the influence of resins on the structural, phase, and dynamic properties of uncured SBR compounds. To this end, compounds containing the highest resin concentration (K45 and D45) were analyzed alongside pure resin samples (Kristalex and Dertoline) and the resin-free compound (REF) for comparison. In order to isolate the effect of the resin and eliminate the influence of cross-linking degree, only uncured compounds were considered.

Qualitative insights were obtained from ^13^C MAS selective experiments, which allow the selective detection of signals from sample moieties with distinct mobility. In addition to ^13^C CP/MAS spectra acquired with an optimized contact time (ct) of 0.5 ms, additional spectra were recorded and compared, including ^13^C CP/MAS spectra with a short ct of 0.05 ms, ^13^C delayed CP/MAS spectra, and ^13^C DE/MAS spectra with a short recycle delay between consecutive scans. In CP spectra with a ct of 0.05 ms, ^13^C signals from rigid domains, characterized by strong ^1^H–^13^C dipolar coupling, are relatively enhanced. Conversely, ^13^C delayed CP and ^13^C DE experiments relatively enhance signals from ^13^C nuclei dipolarly interacting with ^1^H nuclei with spin–spin relaxation time (*T*_2_) > 100 μs and from ^13^C nuclei with short longitudinal relaxation time *T*_1_, respectively, which typically arise from moieties with a high degree of mobility.^43–45^

In Figure 2, the ^13^C selective spectra of K45 and D45 are shown, together with the ^13^C CP/MAS spectra for REF and the pure Kristalex and Dertoline resins, which are included for comparison. In the selective CP spectra of K45 and D45 at the short contact time, the resin signals are enhanced with respect to those from SBR, indicating the rigid character of the resins. In contrast, in the delayed CP spectrum and in the DE spectrum recorded with a short recycle delay, only signals from SBR are visible. This aligns with the high degree of mobility of the polymer chains at the experimental temperature of 298 K, which is far above *T*_g_. In the DE spectra, a signal from TDAE oil is also detected at about 30 ppm (Figure S2).

**Figure 2:**
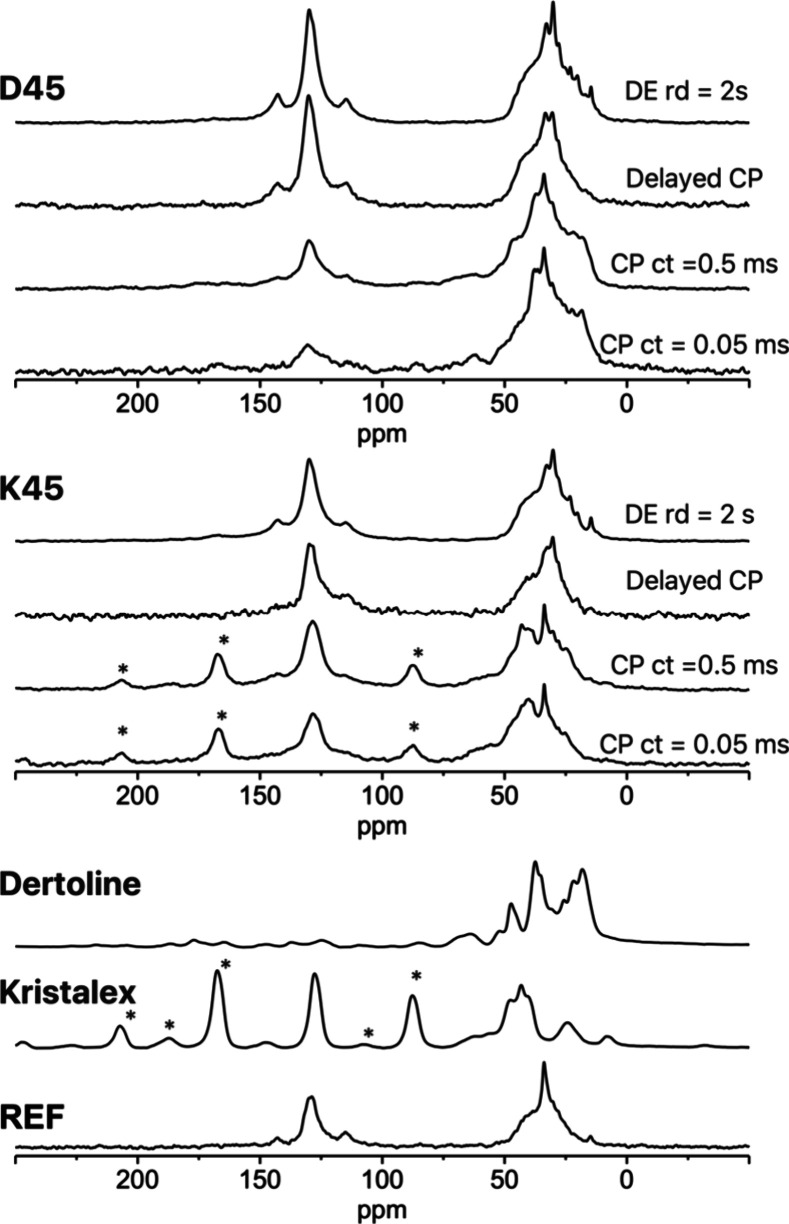
(bottom) ^13^C CP/MAS spectra of REF (ct = 0.5 ms) and of Kristalex and Dertoline resins (ct = 0.4 ms). Middle: ^13^C selective spectra of K45 (CP with ct = 0.5 and 0.05 ms, delayed CP, and DE with rd = 2 s). (top) ^13^C-selective spectra of D45 (CP with ct = 0.5 and 0.05 ms, delayed CP, and DE with rd = 2 s). In all cases, the spinning frequency was 5 kHz. The spinning sidebands are indicated with asterisks. The complete signal assignment of ^13^C resonances for both SBR and the resins is reported in refs 33 and 46.

To obtain more quantitative information on the mobility and proton fraction of distinct domains present in the compounds containing the two resins in different amounts, we analyzed the ^1^H pseudo-FIDs at 303 K of all the uncured samples (Figure S3), obtained by combining the on-resonance ^1^H FIDs acquired using the MSE pulse sequence at short times with the CPMG curves at longer time (>150 μs), as detailed in the Experimental Section. The pseudo-FIDs were modeled using a linear combination of one Gaussian and two exponential decay functions according to the following equation:

4 $$I\left(t\right)=\frac{I\left(0\right)}{100\%}\left(w_{g}e^{-{\left(t/T_{2g}^{*}\right)}^{2}}+w_{e1}e^{-t/T_{2e1}^{*}}+w_{e2}e^{-t/T_{2e2}^{*}}\right)$$

where *w*_i_ represents the weight percentage of the *i*th function, which corresponds to the percentage of the respective proton fraction, while *T*_2i_^*^ denotes its effective transverse relaxation time, which increases monotonically with the degree of mobility.^47^ An example of FID analysis is shown in Figure 3a for K45, while the best-fit parameters obtained for all the samples are reported in Table 2.

**Table 2:** Best-Fit Weights (*w*_i_, %) and Effective Spin–Spin Relaxation Times (*T*_2i_^*^, μs) Obtained from the Analysis of the ^1^H Pseudo-FIDs at 303 K Using the Fitting Function Reported in Equation 4, Along with the Theoretical Percentages of Resin Protons (*w*_H,resin_, %) and Total Rigid Proton Fractions (*w*_H,rigid_, %) Calculated on the Basis of Sample Composition and Values of the Medium *T*_2_^*^ (*T*_2m_^*^, μs) of the Mobile SBR Fraction

sample	*w*_Hresin_	*w*_Hrigid_	*w*_g_	*T*_2g_^*^	*w*_e1_	*T*_2e1_^*^	*w*_e2_	*T*_2e2_^*^	*T*_2m_^*^
REF	–	7	7	17	41	150	52	1250	294
K15	7	13	12	20	53	140	35	1050	217
K25	12	18	16	20	57	140	27	970	192
K35	16	22	22	21	56	135	22	950	179
K45	20	26	25	20	55	120	20	870	154
D15	7	13	14	20	53	200	33	1350	303
D25	12	18	16	18	50	150	34	1100	238
D35	16	22	21	18	46	150	33	1020	233
D45	20	26	22	18	48	115	30	930	175

**Figure 3:**
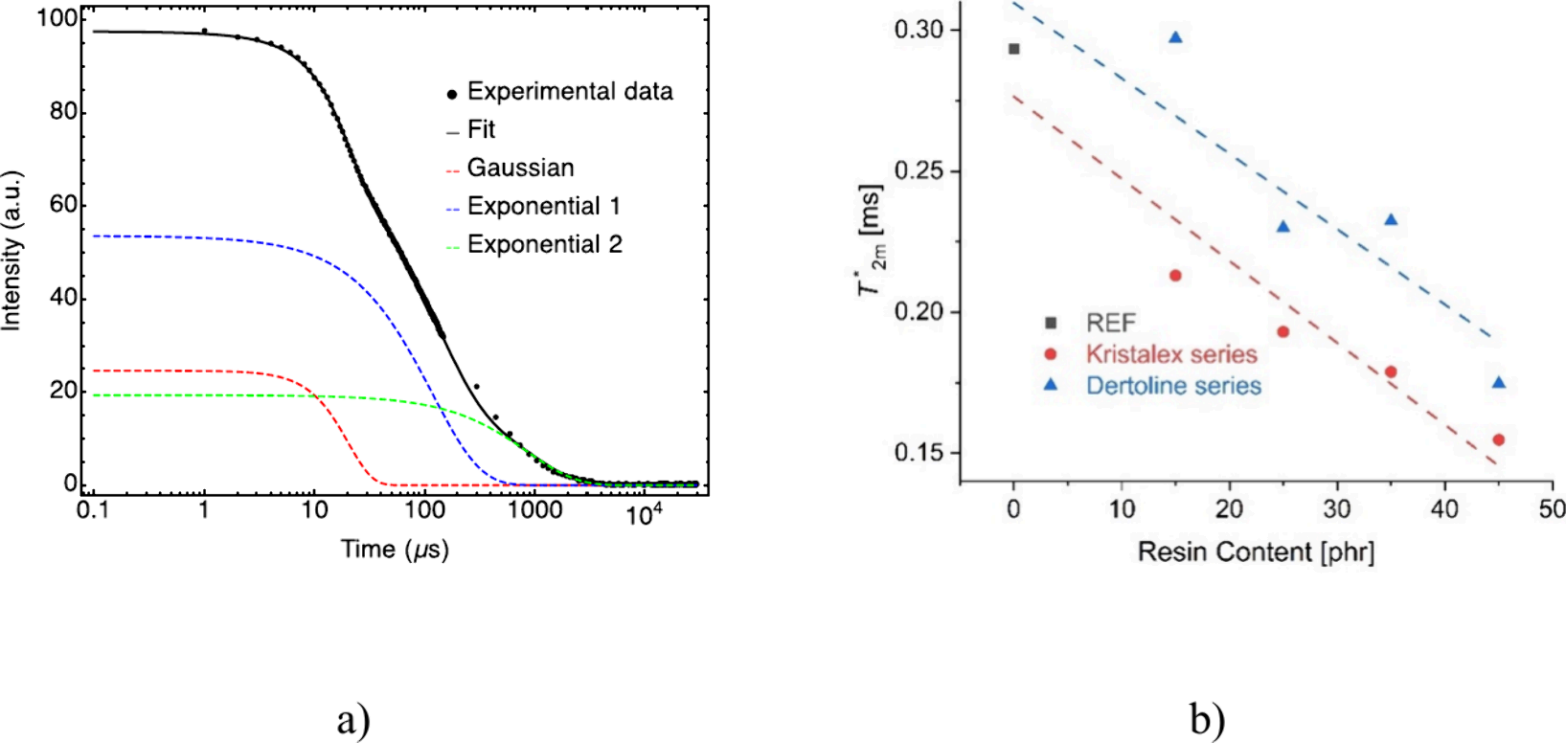
(a) Analysis of the ^1^H pseudo-FID of sample K45 in terms of eq 4. The experimental data are shown as black circles, while the fitting function is represented by the black line; Gaussian, and e1 and e2 exponential functions are shown as red, blue, and green dashed lines. (b) Trends of *T*_2m_^*^ values, calculated as described in the text, as a function of resin content for the compounds containing Kristalex (red circles) and Dertoline (blue triangles) resins and for REF (gray square). The dashed lines represent linear fits to the experimental data.

The Gaussian function is characterized by a *T*_2_^*^ of approximately 20 μs, which is typical of rigid, solid-like domains. Although present in small amounts, this component is already detectable in the SBR reference sample (REF), where it can be attributed to SBR chain segments involved in physical entanglements and/or strongly interacting with filler particles.^33,46^ Interestingly, its weight percentage (*w*_g_) increases monotonically with the resin content, aligning well with the values (*w*_H,rigid_, %) predicted on the basis of the resin amount (Table 2). In particular, *w*_H,rigid_ was estimated as the sum of the contributions from the resin protons and the fraction of “rigid” SBR protons, assumed to be equal to the Gaussian fraction observed in REF. The results confirm the rigid nature of the resins, previously highlighted by ^13^C experiments, even when mixed with SBR, and indicate that no increase in the solid-like SBR fraction occurs upon resin addition.

The exponential components e1 and e2 are characterized by longer *T*_2_^*^ values, which range from 110 to 200 μs for e1 and from 870 to 1350 μs for e2. These values are typical of regions with intermediate to high degrees of mobility, primarily originating from SBR chains and, to a lesser extent, from TDAE oil. The introduction of resin leads to a decrease in the high-mobility fraction (*w*_e2_) and a progressive shortening of *T*_2e1_^*^ and *T*_2e2_^*^ as the resin content increases. This finding is consistent with a reduction in SBR mobility upon resin addition, which leads to a rise in *T*_g_ (*vide infra*). To obtain a clearer picture of the effect of resin on the overall SBR mobility it is useful to look at the medium *T*_2_^*^ value (*T*_2m_^*^) calculated as the inverse of PWRA­(*T*_2_^*^) (section 2.2, eq 1) of the mobile SBR fraction (e1 and e2 components). As shown in Figure 3b, *T*_2m_^*^ decreases linearly by increasing the resin amount in the compound for both resins, due to the slowing down of SBR dynamics.

Finally, for REF, K45, and D45 compounds and pure resins, variable-temperature measurements of ^1^H *T*_1_ and *T*_1ρ_ relaxation times were conducted in the 243–343 K range to investigate the influence of resin on polymer dynamics and to get insights into the degree of mixing between SBR and resin. Indeed, spin diffusion tends to average ^1^H *T*_1_ and *T*_1ρ_ relaxation times of distinct domains, leading to a single value when domain dimensions are smaller than 100–200 Å and 10–20 Å, respectively.^48^

^1^H *T*_1_ data are shown in Figure 4. In the case of REF, ^1^H *T*_1_ decreases monotonically with increasing the temperature, a trend typically associated with relaxation driven by molecular motions with characteristic frequencies lower than the ^1^H Larmor frequency (500 MHz). These motions can be attributed to segmental dynamics linked to the SBR glass transition.^49,50^ Conversely, for the pure resins, an increase in ^1^H *T*_1_ with temperature is observed, suggesting that relaxation is governed by motions with characteristic frequencies higher than the Larmor frequency, likely due to rapid reorientations of resin side groups. For compounds containing 45 phr of resin (K45 and D45), a single *T*_1_ value is measured across the entire temperature range, even at the temperature extremes where the *T*_1_ values of the pure components are mostly different. A similar behavior was already observed for samples with 15 phr of resin. The present findings further support an intimate mixing between SBR and resin on the 100–200 Å scale, even at high resin content, for both Dertoline and Kristalex. In Figure 4, the experimental *T*_1_ values are compared with the medium *T*_1_ (*T*_1m_) calculated as the inverse of PWRA­(*T*_1_) (section 2.2, eq 1), where the different fractions refer to the fractional amount of protons of SBR and resin in the compound and the *T*_1_ values are those of REF and pure resin samples. The deviation of the experimental *T*_1_ values from the *T*_1m_ predictions indicates that dynamics in the resin-containing compounds differs from those of the isolated components, confirming the occurrence of interactions between resin and SBR and dynamics modification upon mixing.

**Figure 4:**
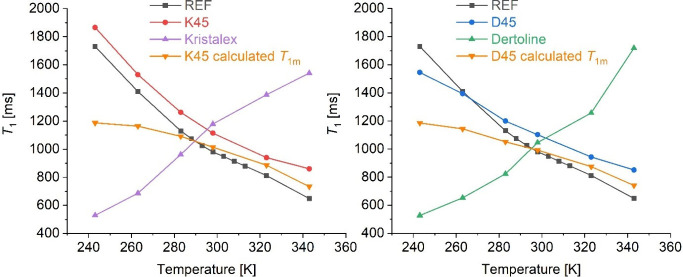
Experimental ^1^H *T*_1_ values of the indicated samples and calculated ^1^H *T*_1m_ for K45 and D45 as functions of temperature.

In the case of ^1^H *T*_1ρ_, the magnetization recovery curves can be reproduced by the linear combination of two or three exponential functions (eq S1). The *T*_1ρ_ values (*T*_1ρ*i*_) and the corresponding weight percentages (*w*_*i*_) of the various components, obtained as best-fit parameters from the analysis of the experimental curves, are reported in Tables S1–S5. However, assigning each component to specific fractions of the sample is not straightforward. For this reason, we calculated a medium *T*_1ρ_ value, *T*_1ρm_, defined as the inverse of PWRA­(*T*_1ρ_) of the different components obtained from the fitting (section 2.2, eq 1). The obtained *T*_1ρm_ values are reported in Tables S1–S5, while their temperature dependences are shown in Figure 5. For REF, the *T*_1ρm_ values are significantly lower than those of the pure resins and display a minimum around 293 K, indicating molecular motions with frequencies comparable to the spin-lock field (46 kHz). As discussed in refs 33,46 , in the investigated temperature range *T*_1ρ_ relaxation is mainly driven by collective dynamics of the polymer chains, often referred to as polymer dynamics, although minor contributions from segmental dynamics cannot be excluded, especially at low temperatures. Samples K45 and D45 show *T*_1ρm_ values and temperature trends similar to those of REF, with the exception of a slight shift of the minimum toward higher temperatures, consistent with a slowdown in polymer motions due to the presence of resins. Notably, in these samples, no long-*T*_1ρ_ components similar to those observed in the pure resin were detected, suggesting intimate mixing between SBR and the resins, in agreement with the *T*_1_ data. However, given the multiexponential nature of the relaxation curves, we can state that homogeneity on a scale of 10–20 Å is not reached.

**Figure 5:**
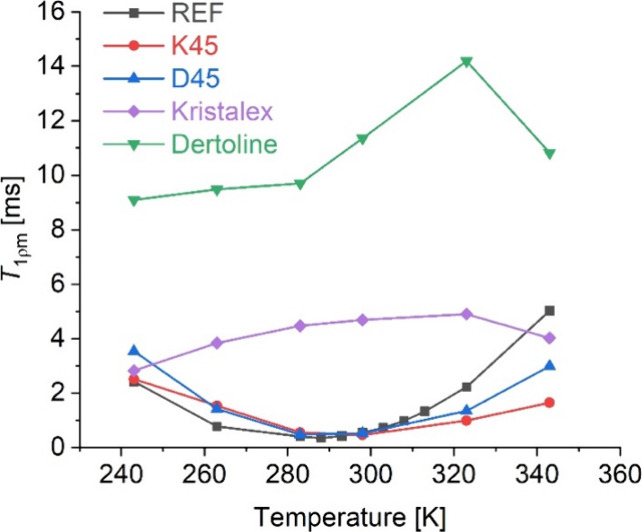
^1^H *T*_1ρm_ values of the indicated samples as functions of temperature.

### 3.2 Determination of the Bound Rubber by Swelling Experiments

Swelling analyses on uncured compounds were performed to determine the bound rubber percentage (BdR), as described in section 2.3. BdR corresponds to the percentage of SBR that remains undissolved after 72 h of swelling in toluene. This fraction is generally considered to represent SBR chains that are either chemically bonded to the filler surface or strongly physisorbed onto it. As reported in Table S6, similar BdR values of approximately 20% were observed for REF and Kristalex-containing samples, with a slight decrease as the resin content increased. In contrast, significantly lower BdR values of about 10% were recorded for the Dertoline-containing samples, approaching the lower detection limit. This suggests that, in the uncured compounds, Dertoline hinders effective interaction between SBR and carbon black. In a previous study, it was reported that the adsorption of resins on the surface of filler particles limited effective polymer–filler interactions.^51^ A similar behavior may be invoked to rationalize the present findings. In particular, the adsorption of Dertoline onto the CB surface could be favored, compared to Kristalex, by physical interactions between the polar groups of the resin and the oxygen-containing functional groups (e.g., phenolic or carboxylic groups) present on the CB surface.^52^ Such interfacial adsorption may effectively screen the filler surface, reducing direct SBR–CB interactions and consequently leading to the lower BdR values observed. However, further investigations, such as transmission electron microscopy, adsorption measurements or systematic studies using CB with different amounts of surface oxygen-containing groups, would be necessary to clarify the mechanism underlying this behavior.

### 3.3 Thermal Behavior and Polymer–Resin Compatibility by DSC

To investigate polymer–resin interactions and compatibility, DSC measurements were performed on both uncured and cured compounds. Representative thermograms are shown in Figure S4. Across the entire temperature range, all samples exhibited a single glass transition around 245–260 K, close to the *T*_g_ of pure SBR. The *T*_g_ values are listed in Table 3. This suggests that the polymer and resin are homogeneously mixed at the nanometer scale, in agreement with ^1^H *T*_1_ and *T*_1ρ_ results. If structural heterogeneity were present, a second transition would be expected near the *T*_g_ of the pure resins, 368 K for Kristalex and 349 K for Dertoline.

**Table 3:** Glass Transition Temperatures Measured by DSC (^DSC^ *T*_g_) and DMA (^DMA^ *T*_g_) for the Uncured and Cured Compounds Reported in K; The Uncertainty Corresponds to the Last Significant Digit

sample	^DSC^ *T*_g_	sample	^DSC^ *T*_g_	^DMA^ *T*_g_
REF	245.3	vREF	250.9	259.7
K15	248.6	vK15	255.1	266.7
K25	250.4	vK25	257.4	270.7
K35	251.7	vK35	257.9	274.2
K45	252.6	vK45	259.1	278.2
D15	247.2	vD15	252.4	262.2
D25	248.2	vD25	253.3	263.2
D35	248.6	vD35	254.4	264.2
D45	249.4	vD45	255.1	265.2

For uncured compounds, *T*_g_ increased consistently with resin content in both the Dertoline and Kristalex series. This is expected, as blending SBR with a higher-*T*_g_ resin raises the overall *T*_g_. For the cured samples, a *T*_g_ increase of approximately 5–7 K with respect to the uncured ones at the corresponding resin content was detected (Table 3). This shift is attributed to the reduced segmental mobility caused by cross-linking (*vide infra*). The *T*_g_ trends with increasing resin content closely follow those observed in the uncured samples. These results suggest that cross-linking does not significantly affect the miscibility or interaction between polymer and resin. However, interpreting the *T*_g_ data in terms of polymer–resin compatibility and miscibility is more complex in the cured systems, as variations in cross-link density introduce additional influencing factors. For this reason, in the rest of the paragraph, we will focus on the *T*_g_ trends obtained for the uncured compounds for a more detailed analysis.

Figure 6 shows the *T*_g_ trends as a function of resin weight percentage relative to the total content of amorphous components (resin, SBR, and TDAE oil). Notably, the experimental data deviate from the ideal behavior predicted by the Fox equation,^53^ which estimates *T*_g_ as the weighted average of the pure components’ *T*_g_ values. Instead, the Gordon–Taylor (GT) model^54^ better fits the observed trends, aligning with previous studies on resin–SBR blends of varying composition.^51^ In particular, our data were fitted to the GT equation:

5 $$T_{g}^{\mathrm{mix}}=\frac{w_{\mathrm{SBR}}T_{g,\mathrm{SBR}}+kw_{R}T_{g,R}}{w_{\mathrm{SBR}}+kw_{R}}$$

where *w*_SBR_ and *w*_R_ are the weight fractions of TDAE-extended SBR and resin, respectively, and *T*_g,SBR_ and *T*_g,R_ are the glass transition temperatures of REF and resin, respectively. The theoretical value of the constant *k*, *k*_0_, is defined as

6 $$k_{0}=\frac{{\Delta C}_{p,R}\left(T_{g,R}\right)}{{\Delta C}_{p,\mathrm{SBR}}\left(T_{g,\mathrm{SBR}}\right)}$$

where Δ*C*_*p*,R_ and Δ*C*_*p*,SBR_ are the variations of heat capacity at *T*_g_ for the pure resin and REF, respectively, measured by DSC. In practice, the *k* parameter in the GT model is typically derived empirically by fitting experimental data. Its deviation from the theoretical value indicates the degree of interaction and compatibility between the mixed components. Figure 6a and Table 4 show the best-fit curves and parameters for both the Dertoline and Kristalex series, alongside the calculated curves with *k* = 1 (Fox model) and *k* = *k*_0_. The best-fit *k* values are significantly lower than *k*_0_, particularly for the Dertoline series. This trend has been observed before and may result from several factors.^51^ One possibility is a reduced effective resin content due to partial interactions between resin and filler particles. This interpretation aligns with the lower bound rubber values observed for resin-containing samples, especially those with Dertoline. Another explanation could be the limited dynamic compatibility between the resin and SBR. While the two phases appear homogeneously mixed at the nanoscale, their segmental mobility may remain partially decoupled, as highlighted by FID analysis.

**Table 4:** Best-Fit Values of the Parameter *k* Obtained from Fitting of *T*_g_ as a Function of the Resin Content for the Uncured Compounds, Along with the Corresponding Theoretical Values (*k*_0_) and the Values of Δ*C*_p,R_ of the Pure Resins (J g^–1^ K^–1^)­^a^

**Sample series**	**Δ** ***C***_**p**,**R**_	***k***_**0**_	***k***
**Dertoline**	0.31	0.46	0.14
**Kristalex**	0.28	0.41	0.21

a The values of *k*_0_ were calculated as reported in the text. To this end, Δ*C*_p,SBR_ = 0.68 J g^–1^ K^–1^ and the values of Δ*C*_p,R_ of the pure resins reported in the table were used, which were determined from the corresponding experimental DSC curves as described in the Experimental Section.

**Figure 6:**
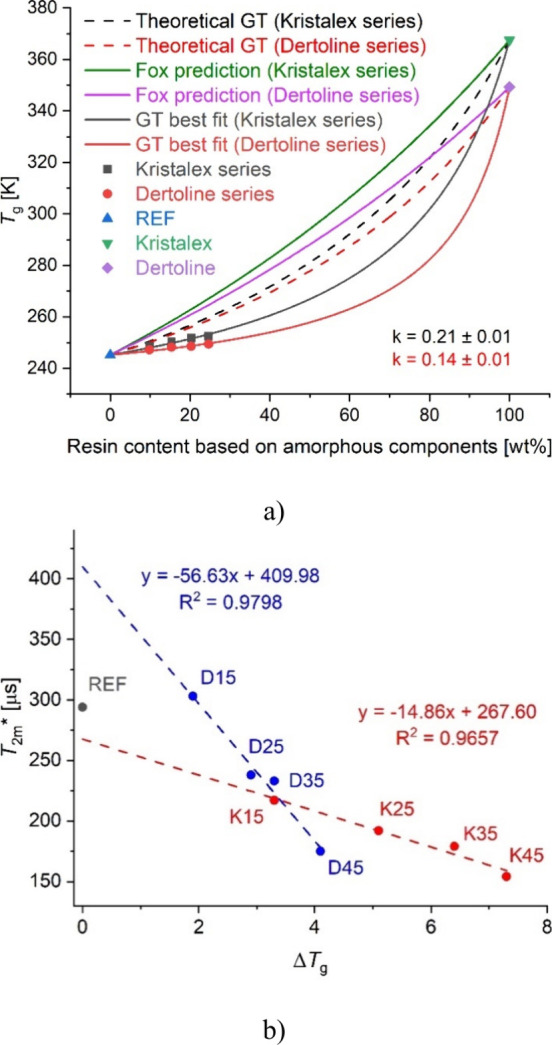
(a) Experimental ^DSC^ *T*_g_ values as functions of resin content for the uncured Kristalex and Dertoline series. Data for the pure resins and the REF sample are also reported. Theoretical trends are based on the GT and Fox equations, while GT best-fit curves highlight differences in resin–polymer interactions. (b) Plots of *T*_2m_^*^ vs Δ*T*_g_ = ^DSC^ *T*_g_ – ^DSC^ *T*_g,SBR_ for the uncured samples.

To gain deeper insights into the origin of the different behavior exhibited by the ^DSC^ *T*_g_ of the compounds in the presence of the two resins, it is interesting to examine the plots of *T*_2m_^*^ obtained from FID analysis as a function of the increase in *T*_g_ with respect to the resin-free compound (Δ*T*_g_ = ^DSC^ *T*_g_ – ^DSC^ *T*_g,SBR_) (Figure 6b). As discussed in section 3.1, *T*_2m_^*^ reflects the average mobility of rubbery SBR domains. These plots therefore provide valuable information on how variations in molecular mobility affect the *T*_g_ of the SBR matrix in the presence of resin. For both resins, a linear decrease of *T*_2m_^*^ with Δ*T*_g_ is observed over the entire composition range. However, differently from Dertoline, for Kristalex the intercept of the linear trend approaches the value of *T*_2m_^*^ of REF, and a much lower slope is observed. These results indicate that the two resins have a different impact on the microstructure and dynamics of the SBR phase. While Kristalex strongly influences the onset of segmental dynamics, as evidenced by the large variation of *T*_g_, Dertoline seems to affect more the chain motions by partially removing physical entanglements (higher intercept) and introducing additional constraints at high resin concentration (steeper slope).

### 3.4 Vulcanization Kinetics and Cross-Link Density by MDR and Swelling Experiments

MDR and swelling experiments were carried out on both CB-filled and CB-free (CF) samples to decouple the effects of filler–rubber and filler–filler interactions from those associated with the resin itself.

The vulcanization behavior was evaluated using a moving die rheometer at 443 K. The resulting MDR rheograms are shown in Figures 7 and S5 for the filled and unfilled compounds, respectively. The kinetic parameters extracted from the experimental curves are summarized in Table 5.

**Figure 7:**
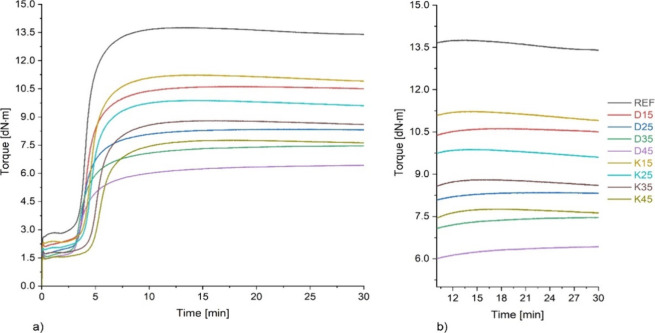
(a) MDR rheograms of the indicated samples at 443 K. (b) Zoom of the postcuring zone (long times). The marching phenomenon is visible for D35 and D45, while REF and the Kristalex-containing samples display a slight reversion.

**Table 5:** Parameters Obtained from MDR Analysis^a^

sample	*M*_H_	*M*_L_	*M*	*t*_s2_	*t*_c90_	*CRI*	*CT*	*MMI*	*M*_c_^–1^
REF	13.76	2.61	11.15	3.68	6.13	0.68	5.94	n.o.	1.59
K15	11.23	2.28	8.95	4.23	6.82	0.64	6.54	n.o.	1.15
K25	9.88	1.94	7.94	4.30	6.88	0.65	6.73	n.o.	1.04
K35	8.81	1.72	7.09	4.93	7.76	0.59	7.26	n.o.	0.92
K45	7.76	1.43	6.33	5.24	8.35	0.54	7.61	n.o.	0.85
D15	10.62	2.10	8.52	3.89	6.85	0.56	6.00	n.o.	0.85
D25	8.35	1.71	6.64	3.73	7.08	0.50	5.78	n.o.	0.63
D35	7.47	1.57	5.90	3.55	8.16	0.36	5.65	0.010	0.57
D45	6.44	1.45	4.99	3.70	9.28	0.30	5.60	0.013	0.52
REF_CF	5.84	0.97	4.87	11.23	15.97	0.35	14.2	n.o.	1.74
K15_CF	4.78	0.73	4.05	10.51	14.55	0.41	13.3	n.o.	1.38
K45_CF	3.32	0.54	2.78	14.95	18.19	0.51	16.9	n.o.	1.04
D15_CF	4.36	0.69	3.67	8.25	11.77	0.47	10.7	n.o.	1.14
D45_CF	2.86	0.47	2.39	9.91	11.85	0.86	9.4	0.007	0.81

a *M*_H_, *M*_L_, and *M* are given in units of dN·m; *t*_s2_, *t*_c90_, and *CT* are in min; *CRI* is in s^–1^; marching modulus intensity (*MMI*) is in dN·m/min. Values of the cross-linking degree (*M*_c_^–1^, 10^–5^ mol/g) determined by swelling experiments are also reported. The code n.o. means that marching effect was not observed.

For the CB-filled samples, following the scorch time (*t*_s2_), during which reactive curing species are generated, a rapid increase in torque is observed, marking the onset of the cross-linking process.^55^ The reference point for the completion of vulcanization is defined as *t*_c90_, which corresponds to the time at which the torque reaches 90% of the difference between the minimum and maximum values. For the Kristalex series, both the onset and the completion of vulcanization are delayed compared to the REF sample, as evidenced by the increasing *t*_s2_ and *t*_c90_ values with higher resin content. Furthermore, a decrease in cure rate index (*CRI*, eq 3) occurs upon resin addition. A reversion phenomenon is observed at long curing times in all Kristalex-containing compounds, similarly to the REF sample. In contrast, the Dertoline series displays values of *t*_s2_ similar to REF without significant variations with resin content. However, for D35 and D45 a pronounced marching effect^56^ at long curing times is observed, characterized by a continuous increase in torque without reaching a stable plateau, as highlighted in the zoomed postcure region of Figure 7b. Due to this marching behavior, conventional parameters such as *t*_c90_ and *CRI* become less reliable. To overcome this limitation and achieve a more accurate evaluation of the curing kinetics, the cure time (*CT*) values were determined from the differential curves of the rheograms,^39^ as described in Section 2.5. The *CT* values reported in Table 5 clearly reveal the retarding effect of Kristalex and the mild accelerating effect of Dertoline, both of which become more pronounced with increasing the resin content. The slowdown of curing induced by Kristalex is consistent with (i) the occurrence of interactions between the resin and curatives, potentially lowering the effective availability of the curatives to SBR, and (ii) an increase in compound viscosity related to the high softening point of the resin, which together could restrict diffusion-controlled stages of vulcanization. The accelerated curing behavior observed with Dertoline may result from a combination of chemical and physical contributions. Polar functionalities in the rosin-based resin may influence the ZnO–accelerator equilibrium,^57^ while a concomitant reduction in filler–polymer interactions, as evidenced by BdR measurements, could enhance polymer chain and curative mobility, thereby promoting a more rapid development of the cross-linked network during the initial stages of vulcanization. These results align with previous observations on SBR compounds containing 15 phr of resin.^33^

In the absence of carbon black, a slowdown of vulcanization kinetics is observed (Figure S5 and Table 5), consistent with the catalytic role of CB in sulfur curing of SBR previously reported in the literature.^58^ However, the trends of the kinetic parameters as a function of resin content are retained in the CB-free series as well.

The observation of marching behavior at high Dertoline loadings warrants further discussion, as it may provide insight into the role of the resin in the vulcanization process. For samples showing the marching effect, the marching module intensity (*MMI*) was calculated as the slope in the last part of the MDR curve:

$$\mathrm{MMI}=\frac{\mathrm{torque}\left(30\;\min\right)-\mathrm{torque}\left(15\;\min\right)}{30\;\min-15\;\min}$$ 7

where torque­(30 min) and torque­(15 min) are the torque values at the longest curing time (30 min) and at 15 min, respectively. The obtained values are reported in Table 5. The increase in torque at extended curing times may arise from two concomitant effects. First, the development of a filler–filler network, facilitated by weaker rubber–filler interactions, may contribute to the progressive increase in stiffness. Second, interactions between the accelerator CBS and the polar functional groups of the resin could modify the effective sulfur-to-accelerator ratio. An increase in this ratio has been reported as a primary cause of marching behavior in silica-filled SBR compounds,^59^ where protracted cross-linking was attributed to the partial adsorption of CBS on silica particles. In our samples, the contribution of CBS–resin interactions is supported by the occurrence of observable marching effect even in the absence of carbon black, as reported in Table 5 (*MMI* = 0.007 dN·m/min for D45_CF). However, the significantly higher *MMI* observed for the CB-filled compound (*MMI* = 0.013 dN·m/min for D45) indicates that filler-related effects further enhance the protracted network development, suggesting that both mechanisms operate concurrently.

As shown in Figure 7 and Table 5, both resins lead to a reduction in the minimum torque (*M*_L_) at short curing times, more pronounced for Dertoline, with this effect increasing as the resin content rises. This behavior suggests that both Kristalex and Dertoline act as plasticizers at the vulcanization temperature, enhancing processability and promoting better mixing of the compound components, in line with previous findings.^33^ It is well established that the difference between the maximum torque (*M*_H_) and *M*_L_, denoted as *M*, is directly related to the cross-link density.^60^ The addition of resin results in a decrease in *M*, which can be primarily attributed to a reduction in vulcanization efficiency. Also in this case, the effect is more evident for the Dertoline series with a less pronounced difference between the two resins in the absence of CB. This is likely due to the dilution of the SBR matrix and, possibly, to interaction occurring between the curatives and the resin, which may reduce the availability of curatives for vulcanization. It should be noted that torque values are also affected by the plasticizing effect of the resin at elevated temperatures^61^ and, in filled compounds, by filler reinforcement and the formation of a rubber–filler network. Accordingly, lower torque values are observed for the CB-free samples.

To obtain a direct measurement of cross-link density, swelling experiments were conducted following the Flory–Rehner method.^62^ The cross-link density values (*M*_c_^–1^) derived from equilibrium swelling are reported in Table 5. A clear trend is observed for both CB-filled and CB-free samples: *M*_c_^–1^ decreases as the resin content increases. This effect is more pronounced for Dertoline compared to Kristalex, likely due to stronger interaction between the curatives and the polar functional groups in Dertoline. As a result, the decrease in *M* observed in MDR curves in the presence of resin is primarily due to a lower cross-link density. A linear relationship is found between cross-link density and *M*, but with different slopes depending on the resin type and the presence of CB (Figure 8). In particular, for CB-filled compounds the Dertoline series displays a steeper slope than the Kristalex series, and shows a more pronounced deviation from the reference (REF). This indicates that, for the same cross-link density, the *M* values are higher in Dertoline-containing samples compared to those with Kristalex. This behavior is likely attributable to an increase in viscosity induced by Dertoline at the experimental temperature, stemming from factors other than cross-linking. To this respect, a comparison with the trends observed for the series of CB-free samples is informative. Although only two resin concentrations are available for these systems, the *M* versus cross-link density plots of both Kristalex and Dertoline series exhibit a lower slope compared to the corresponding CB-filled samples, and the slopes observed for the two resins are quite similar. This suggests that, in the absence of CB, the effect of resin content on *M* is less pronounced and mainly governed by changes in the polymer network itself. Conversely, in the CB-filled samples, the stronger dependence of *M* on resin content may reflect changes in the development of filler–filler and filler–rubber networks as a function of resin loading. The higher slope observed for the Dertoline series compared to the Kristalex one could arise from different specific interactions. In particular, the weaker filler–rubber interaction in the presence of Dertoline, as indicated by the lower BdR values (section 3.2 and Table S6), might promote filler aggregation and the formation of a more extended filler–filler network.

**Figure 8:**
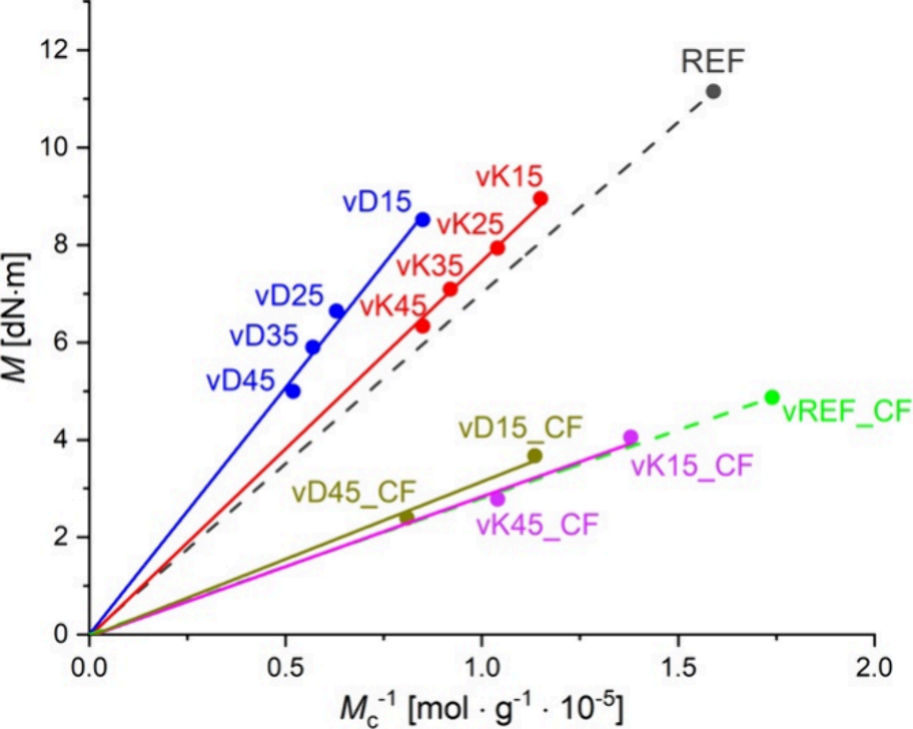
Plot of *M* derived from MDR curves vs cross-link density (*M*_c_^–1^) from swelling experiments for vREF (black), vREF_CF (green), Dertoline series with CB (blue) and without CB (ocher), and Kristalex series with CB (red) and without CB (magenta). Linear fits (solid lines) of experimental data are also shown.

### 3.5 Mechanical Properties

Tensile strength tests provided insights into the influence of the different resins on the tensile properties of vulcanized SBR compounds. The stress–strain curves of CB-filled and CB-free samples are shown in Figures 9 and S6, respectively, while the moduli at different elongations (*M*_10_, *M*_20_, *M*_50_, *M*_100_, *M*_200_, and *M*_300_), tensile strength at break (*TS*_b_), and elongation at break (*E*_b_) are summarized in Table 6 for all samples.

**Figure 9:**
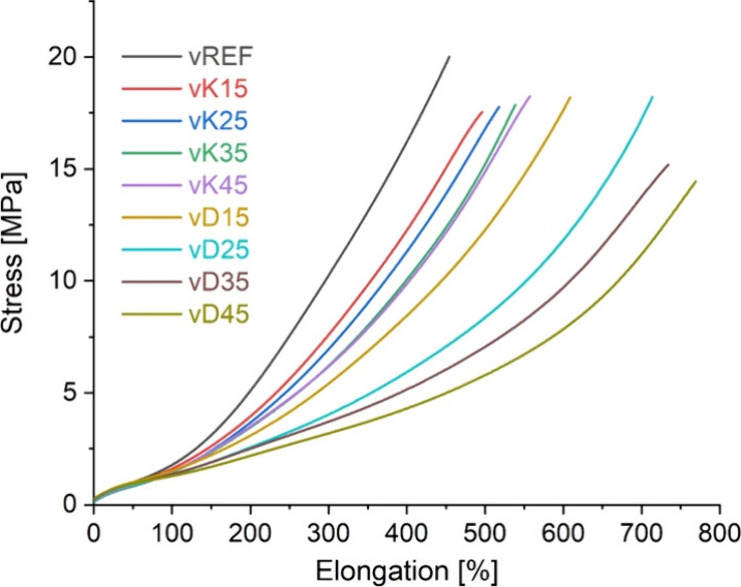
Stress–strain curves of the indicated samples.

**Table 6:** Parameters Obtained from the Stress–Strain Curves of the Indicated Samples^a^

sample	*M*_10_	*M*_20_	*M*_50_	*M*_100_	*M*_200_	*M*_300_	*TS*_b_	*E*_b_
vREF	0.41	0.59	1.00	1.79	5.10	10.27	19.87	445
vK15	0.37	0.53	0.91	1.62	3.96	7.59	17.38	484
vK25	0.36	0.50	0.83	1.44	3.66	6.99	17.38	514
vK35	0.37	0.51	0.83	1.44	3.45	6.21	17.26	532
vK45	0.42	0.57	0.89	1.50	3.50	6.19	17.52	540
vD15	0.50	0.67	1.00	1.53	3.09	5.42	17.95	605
vD25	0.46	0.61	0.89	1.33	2.56	4.04	17.61	705
vD35	0.50	0.66	0.93	1.37	2.53	3.76	14.91	714
vD45	0.56	0.70	0.93	1.28	2.20	3.19	14.18	760
vREF_CF	0.19	0.27	0.43	0.63	1.09	–	1.56	263
vK15_CF	0.18	0.25	0.39	0.56	0.92	1.59	2.08	329
vK45_CF	0.19	0.25	0.38	0.52	0.83	1.49	5.21	434
vD15_CF	0.18	0.25	0.41	0.59	0.98	1.66	2.80	403
vD45_CF	0.19	0.27	0.43	0.60	0.99	1.64	7.23	610

a Moduli at 10%, 20%, 50%, 100%, 200%, and 300% elongation (*M*_10_, *M*_20_, *M*_50_, *M*_100_, *M*_200_, and *M*_300_) are given in MPa; tensile strength at break (*TS*_b_) is in MPa; elongation at break (*E*_b_) is in %.

For both the Dertoline and Kristalex CB-filled samples (Figure 9 and Table 6), the stress–strain curves of the resin-containing compounds lie below that of the vREF sample, indicating that lower stress is required to achieve the same level of elongation in the presence of resin. This softening effect becomes progressively more pronounced with increasing resin content and is considerably more evident for Dertoline than for Kristalex at all concentrations. The introduction of resin leads to an increase in *E*_b_ and a decrease in *TS*_b_, more pronounced for Dertoline. By increasing the resin content, *E*_b_ steadily increases while *TS*_b_ remains almost constant for Kristalex and decreases for Dertoline. These trends can be ascribed to the decrease of cross-link density observed by increasing resin content (Table 5), as well as to mechanical plasticization induced by the presence of resin. Interestingly, comparing samples vK45 and vD15, which show the same cross-link density value, it is evident that Dertoline has a higher mechanical plasticizing effect. This behavior may also arise from the reduced filler–rubber interaction in the Dertoline-containing samples, as demonstrated by the lower BdR percentage for vD15 (10%) compared to vK45 (19%) (Section 3.2 and Table S6). The observed behavior is consistent with a previous study on SBR compounds containing 15 phr Dertoline and Kristalex.^33^ However, when comparing samples with identical compositions, the compounds investigated in the present work exhibit lower stress values at comparable elongation levels and reduced *TS*_b_. This difference may be attributed to the distinct mixing methods employed in the two studies and/or experimental variability.

Different results are obtained for the CB-free series (Figure S6 and Table 6). Indeed, in the absence of filler, the incorporation of either Kristalex or Dertoline does not significantly affect the stress values up to about 200% elongation. The progressive decrease in stress observed with increasing resin content for the CB-filled samples should therefore arise from filler–filler and filler–rubber interactions, which are clearly influenced by both the type and the amount of resin. Moreover, in the CB-free compounds, the addition of both resins leads to a progressive increase in both *TS*_b_ and *E*_b_. These effects are slightly more pronounced for Dertoline than for Kristalex. The increase in *E*_b_ in the absence of CB confirms that this parameter is primarily governed by the reduced cross-link density and by the mechanical plasticizing effect of the resin, more evident for Dertoline, rather than by filler dispersion effects. The opposite trends of *TS*_b_ in filled and unfilled samples indicates that this property is strongly affected by filler–filler and filler–rubber interactions. Interestingly, in the absence of CB, both resins contribute to improved resistance at high elongation, possibly acting as reinforcing agents at large deformation.

### 3.6 Viscoelastic Behavior

The viscoelastic behavior of cured CB-filled compounds was investigated by single-frequency DMA measurements carried out in the temperature range from 193 to 303 K. The DMA curves of all cured compounds are shown in Figure 10. These results offer insights into how different resins influence the mechanical and dynamic properties of SBR compounds.

**Figure 10:**
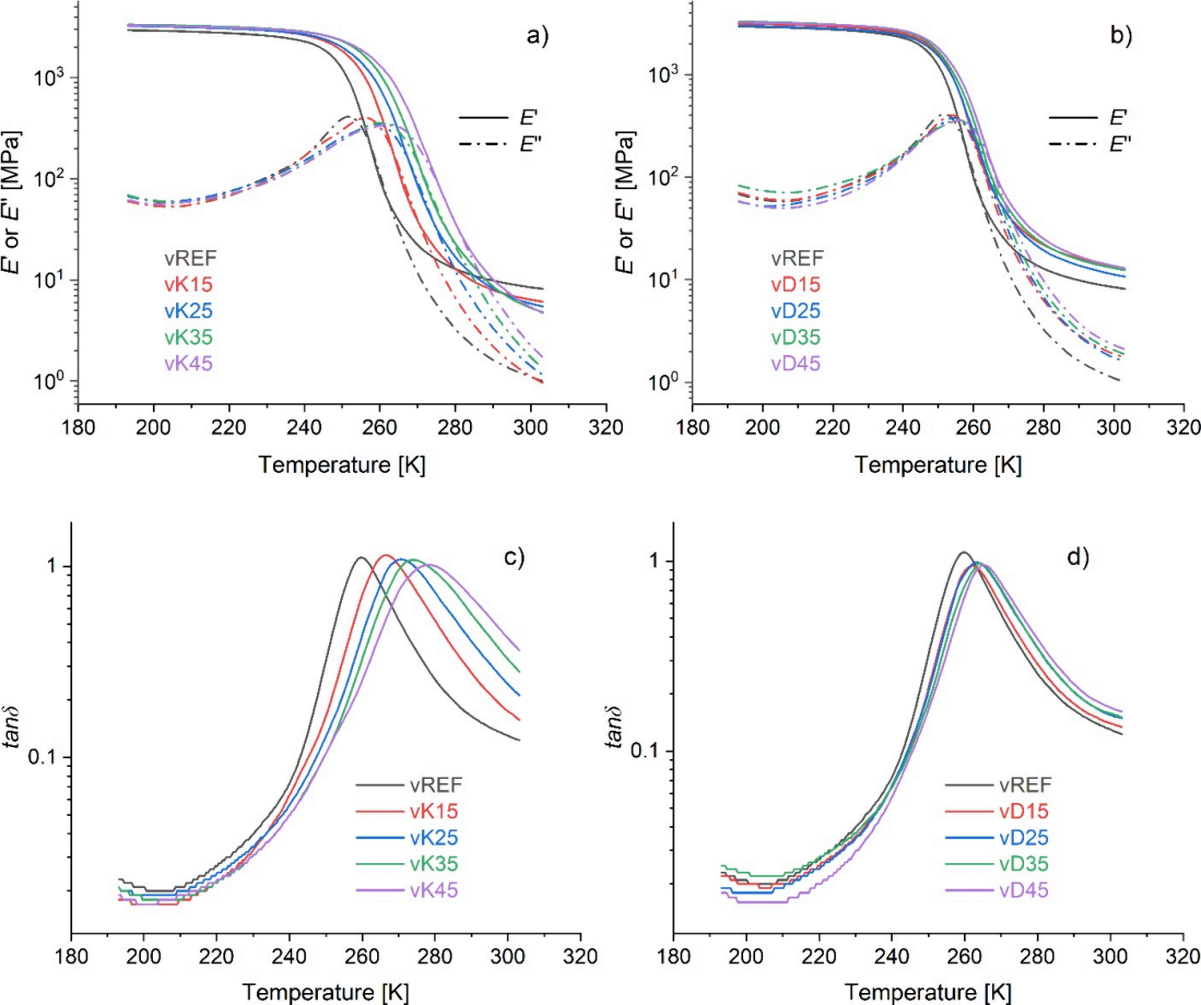
Temperature-sweep DMA curves for the Kristalex and Dertoline series of CB-filled samples: (a, b) storage modulus (*E*′) and loss modulus (*E*″); (c, d) loss factor (*tan*δ).

A steep drop in the elastic modulus (*E*′) and a concomitant increase of the loss modulus (*E*″) occur between 253 and 293 K, corresponding to the glass transition (Figure 10a,b). Upon resin addition and by increasing the resin content, this transition shifts to higher temperatures, consistent with the *T*_g_ increase observed in DSC measurements. Moreover, below the glass transition temperature of the compounds, i.e., when the polymer matrix is in the glassy state, an increase in *E′* is observed for the resin-containing samples compared to REF, indicating an overall stiffening effect. *E″*, on the other hand, does not show significant changes in the case of Kristalex, while it exhibits an irregular trend for Dertoline, suggesting a complex interaction with the polymer matrix. Above the glass transition temperature of the compounds, i.e., in the rubbery state of the polymer matrix, the effect of the resins on *E′* clearly depends on the type of resin and shows an irregular trend with resin concentration. Kristalex generally causes a reduction in *E′* compared to REF, likely due to resin-induced plasticization and/or to a dilution effect of the polymer matrix that reduces its load transfer capability.^63^ Conversely, the incorporation of Dertoline results in an increase in *E′*, indicating that, in this case, the stiffening effect is predominant. This behavior may be tentatively associated with a less effective filler dispersion and the consequent development of a more extended filler–filler network in the presence of Dertoline, as suggested by BdR measurements (Section 3.2). For both resins, *E″* increases with their incorporation and with increasing resin content, a trend that may be attributed to a reduced viscosity of the matrix, consistent with a plasticizing effect of the resin above *T*_g_, together with additional dissipative contributions arising from the increased interfacial areas within the composite.

A clearer assessment of the effect of resin on the viscoelastic behavior of SBR compounds can be achieved moving to the loss factor (*tan*δ) representation of the DMA curves. Indeed, *tan*δ provides a direct indication on the balance between dissipative and elastic behavior. All the *tan*δ curves of the investigated samples (Figure 10c,d) show a maximum at the glass transition. The *T*_g_ values from DMA (^DMA^ *T*_g_), determined as the temperature corresponding to the maximum of the *tan*δ peak, are listed in Table 3. The ^DMA^ *T*_g_ moves to higher temperatures upon resin addition and regularly increases by increasing the resin content. As expected, the extent of this shift depends on the resin type.^64^ In the Kristalex series, increasing resin content shifts the *tan*δ peak to higher temperatures and broadens the transition, indicating good compatibility and strong interaction between resin and SBR.^61^ It is also important to consider that variations in cross-link density may contribute to differences in *T*_g_. In contrast, Dertoline-containing samples exhibit only a modest shift of the *tan*δ peak compared to the reference, whose position remains nearly unaffected by the resin content. This behavior reflects the limited *T*_g_ variations also observed by DSC. Moreover, the reduced amplitude of the *tan*δ peak in the presence of Dertoline indicates a lower damping capacity and, consequently, a higher stiffness relative to Kristalex, in agreement with the trends observed in the E′ curves. This effect can be ascribed to both the inherently lower *T*_g_ of Dertoline and its lower compatibility with the SBR matrix, as also suggested by the GT analysis of the DSC results (see section 3.3).

Finally, it is interesting to examine the *tan*δ values at 273 and 303 K (Figure 11), which are commonly associated with wet grip and rolling resistance, respectively.^18,19^ For both resins, *tan*δ at 273 K increases with resin content, indicating an enhancement in wet grip performance. This increase is more pronounced in the case of Kristalex, likely due to its glass transition temperature being closer to the measurement temperature. At 303 K, Kristalex induces a significant rise in *tan*δ, suggesting a potential increase in rolling resistance, whereas Dertoline has a negligible influence on this parameter. These observations point to complementary effects of the two resins on tire performance: Kristalex enhances wet grip, particularly beneficial under icy or wet conditions, while Dertoline minimizes impact on rolling resistance, which is advantageous for tire longevity and fuel efficiency. However, it should be noted that the samples analyzed in this study are model compounds, not intended for real-life applications. Furthermore, the observed trends might be influenced by the proximity of the test temperatures to the glass transition temperature of the compounds. Therefore, further investigation on technologically relevant systems is necessary to validate these findings.

**Figure 11:**
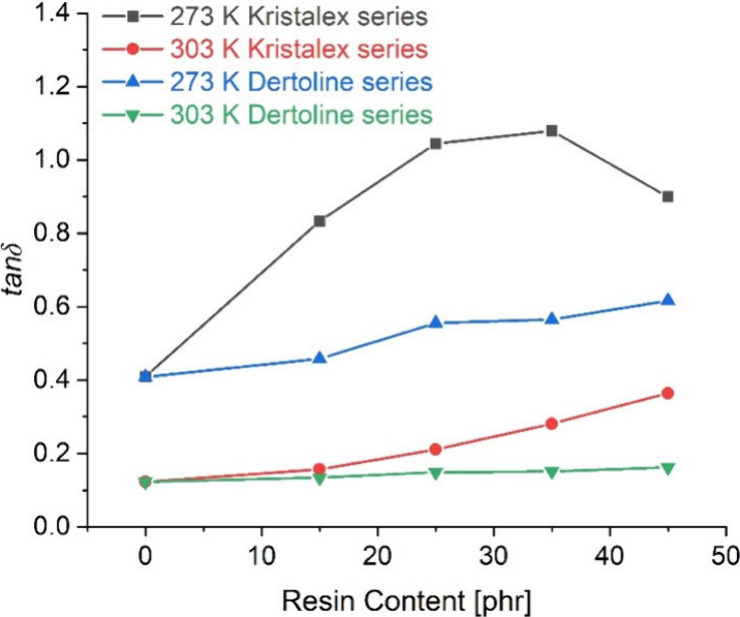
Plots of *tan*δ of CB-filled samples at 273 and 303 K as functions of resin content.

## 4 Conclusions

This study provides a systematic multiscale analysis of the role of tackifying resin type and concentration on the structural, dynamic, and mechanical behavior of SBR compounds relevant to tire applications. The integration of complementary techniques spanning from solid-state NMR to macroscopic mechanical testing enabled a correlation between molecular-level properties and bulk performance.

While good polymer–resin miscibility was observed at the nanoscale for all formulations, the two resins induce markedly different effects on molecular mobility, rubber–filler interactions, and vulcanization kinetics. In particular, Dertoline reduces effective SBR-CB interactions, promotes filler aggregation at high loadings, and significantly alters the curing behavior through specific interactions with the components of the curing package, ultimately leading to a marching behavior and a reduced cross-link density. Kristalex, in contrast, primarily acts as a vulcanization retarder, likely affecting the availability or diffusion of curing agents. These differences translate into distinct modifications of the macroscopic mechanical and viscoelastic responses, with implications for key tire performance indicators such as wet grip and rolling resistance.

Overall, the results demonstrate that resin chemistry plays a technologically relevant role in governing multiscale structure–property relationships in rubber compounds. Further systematic investigations aimed at elucidating the underlying mechanisms will be essential to guide formulation strategies and support the effective implementation of bio-based resins in next-generation sustainable tire materials.

## Supplementary Material



## Data Availability

The data presented in this study are available on request from the corresponding authors.

## Notes

Example of the determination of the cure time from MDR experiments (Section S1); expansions of high-resolution ^13^C SSNMR spectra of D45, ^1^H pseudo-FIDs of CB-filled samples, and ^1^H T_1ρ_ analysis (Section S2); bound rubber measurements (Section S3); representative DSC curves (Section S4); MDR rehograms of CB-free samples (Section S5); stress–strain curves of CB-free samples (Section S6) (PDF)
