# Supplementary material for: Toward Greener Rubber: Impact of Resin Type and Amount on Curing, Network Structure, and Viscoelastic Properties in SBR compounds
Source: ACS Polym Au. 2026 Mar 19;6(3):891–905. doi: 10.1021/acspolymersau.6c00018 (PMC13261731; doi:10.1021/acspolymersau.6c00018)
Supplement: Supplementary file 1 [file lg6c00018_si_001.pdf]

Towards Greener Rubber: Impact of Resin Type and Amount on Curing, Network Structure, and Viscoelastic Properties in SBR compounds

Michele Pierigé,[1] Francesca Nardelli,[1,2,3,*] Mattia Cettolin,[4] Andrea Causa,[4] Luca Giannini,[4] Francesca Martini,[1,2,3,*] Lucia Calucci,[2,3] Marco Geppi[1,2,3]

[1]*Dipartimento di Chimica e Chimica Industriale, Università di Pisa, Via G. Moruzzi 13, 56124 Pisa (Italy)*
[2]*Istituto di Chimica dei Composti OrganoMetallici, Consiglio Nazionale delle Ricerche, Via G. Moruzzi 1, 56124 Pisa (Italy)*
[3]*Centro per l'Integrazione della Strumentazione Scientifica dell'Università di Pisa (CISUP), Lungarno Pacinotti 43/44, 56126 Pisa, Italy*
[4]*Pirelli Tyre SpA, Viale Sarca 222, 20126 Milano, Italy*

[*]**Corresponding Authors:** *Francesca Martini, email: francesca.martini@unipi.it; Francesca Nardelli, email: francesca.nardelli@unipi.it*

Table of Contents

S1. MDR experiments *S2*
S2. SSNMR experiments *S3*
  S2.1 High-resolution ^{13}C SSNMR spectra *S3*
  S2.2 ^{1}H FID analysis *S3*
  S2.3 ^{1}H $T_{1\varrho}$ analysis *S4*
S3. Bound Rubber *S6*
S4. DSC experiments *S7*
S5. MDR experiments *S8*
S6. Stress-strain experiments *S9*

S1. MDR experiments

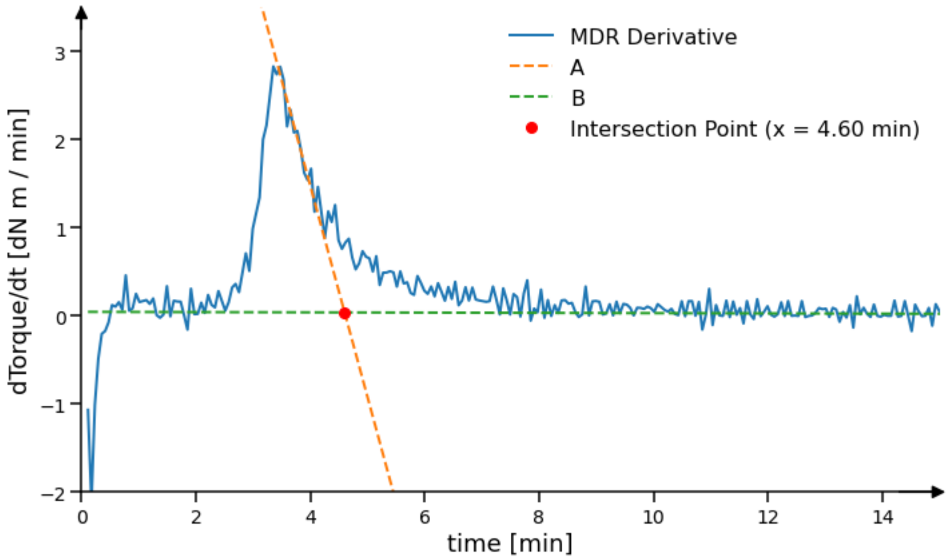

Figure S1. Determination of the cure time (CT) as the intersection point between the A and B tangential lines to the MDR derivative curve plus one additional min. The graph shows the analysis carried out for the D45 sample, for which a CT of 5.6 min was determined.

S2. SSNMR experiments

S2.1 High-resolution ${}^{13}C$ SSNMR spectra

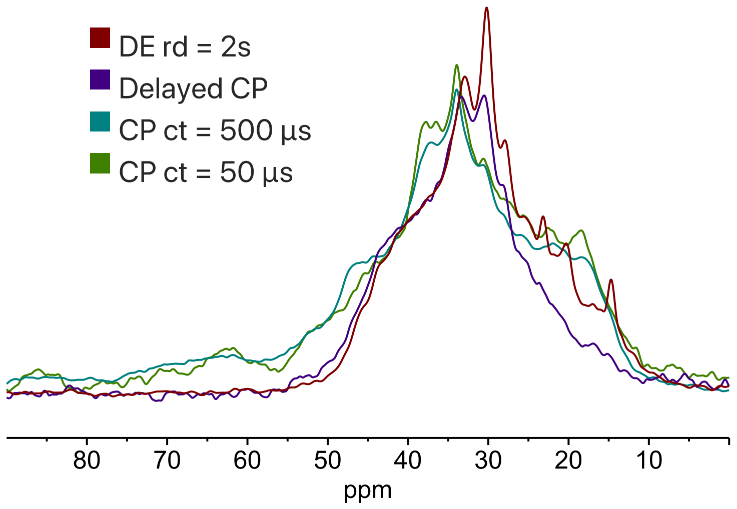

Figure S2. Expansions of the indicated ${}^{13}C$ spectra of D45.

S2.2 ${}^{1}H$ FID analysis

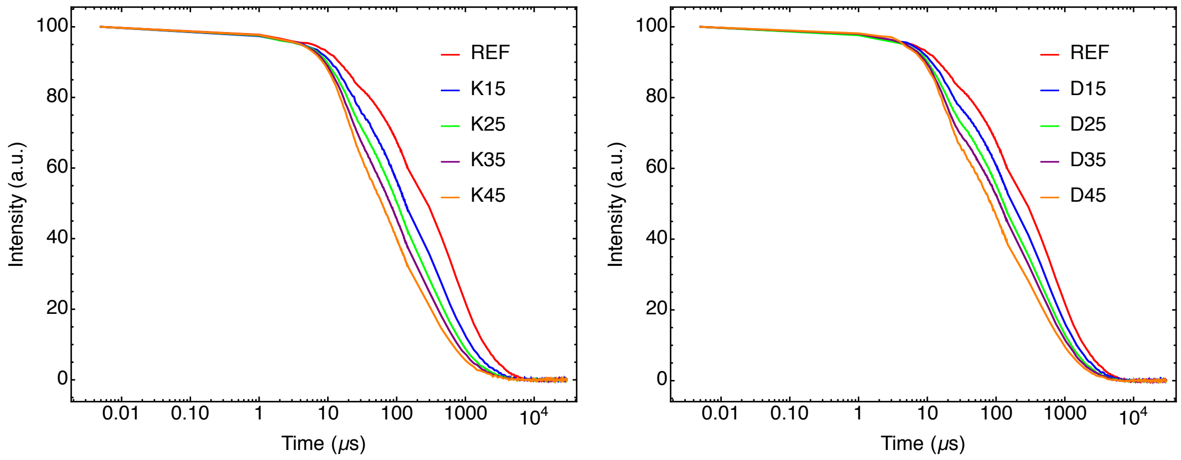

Figure S3. ${}^{1}H$ pseudo-FIDs at 303 K of the indicated uncured compounds of the Kristalex (left) and Dertoline (right) series.

S2.3 ^1H $T_{1\varrho}$ analysis

The experimental $T_{1\varrho}$ decay curves were fitted with a linear combination of two or three exponential functions, according to the general equation:

$$I(t) = \frac{I(0)}{100}\sum_{i=1}^{n} w_i e^{-\frac{t}{T_{1\rho i}}} \quad \text{(S1)}$$

Where $I(0)$ is the signal intensity at $t = 0$ and w_i and $T_{1\varrho i}$ are the weight percentage and $T_{1\varrho}$ value of the i-th component. The best-fit parameters obtained for Kristalex, Dertoline, REF, K45 and D45 are reported in Tables S1-S5.

Table S1. Best-fit parameters from the analysis of Kristalex $T_{1\varrho}$ magnetization recovery curves, fitted using a sum of two exponential functions according to Equation S1. Reported values are the relaxation times $T_{1\varrho i}$ (ms), the corresponding weight percentages w_i, and the medium relaxation $T_{1\varrho m}$ calculated as the inverse of PWRA.

T (K)	w_1 (%)	w_2 (%)	$T_{1\varrho 1}$ (ms)	$T_{1\varrho 2}$(ms)	$T_{1\varrho m}$ (ms)
243	9	91	0.6	4.5	2.8
263	10	90	1.0	5.7	3.8
283	7	93	0.8	6.7	4.5
298	7	93	0.8	7.6	4.7
323	7	93	0.9	7.6	4.9
343	10	90	0.8	7.2	4.0

Table S2. Best-fit parameters from the analysis of Dertoline $T_{1\varrho}$ magnetization recovery curves, fitted using a sum of two exponential functions according to Equation S1. Reported values are the relaxation times $T_{1\varrho i}$ (ms), the corresponding weight percentages w_i, and the medium relaxation $T_{1\varrho m}$ calculated as the inverse of PWRA.

T (K)	w_1 (%)	w_2 (%)	$T_{1\varrho 1}$ (ms)	$T_{1\varrho 2}$ (ms)	$T_{1\varrho m}$ (ms)
243	8	92	1.9	13.3	9.1
263	7	93	1.7	14.3	9.5
283	6	94	1.3	16.6	9.7
298	5	95	1.3	20.2	11.4
323	5	95	1.5	26.7	14.2
343	6	94	1.1	25.1	10.8

Table S3. Best-fit parameters from the analysis of REF $T_{1\varrho}$ magnetization recovery curves, fitted using a sum of three exponential functions according to Equation S1. Reported values are the relaxation times $T_{1\varrho i}$ (ms), the corresponding weight percentages w_i, and the medium relaxation $T_{1\varrho m}$ calculated as the inverse of PWRA.

T (K)	w_1 (%)	w_2 (%)	w_3 (%)	$T_{1\varrho 1}$ (ms)	$T_{1\varrho 2}$ (ms)	$T_{1\varrho 3}$ (ms)	$T_{1\varrho m}$ (ms)
243	10	72	18	0.5	3.6	10.0	2.4
263	21	72	7	0.4	1.2	10.1	0.8
283	88	8	4	0.4	2.5	10.1	0.4
288	49	45	6	0.3	0.8	10.0	0.4
293	45	48	7	0.3	1.0	10.0	0.4
298	41	51	8	0.3	1.2	10.0	0.6
303	35	55	10	0.4	1.5	10.0	0.7
308	30	56	14	0.5	1.8	10.0	1.0
313	24	58	19	0.5	2.1	10.4	1.3
323	14	54	32	0.6	2.8	11.7	2.2
343	8	49	43	1.0	5.2	21.9	5.0

Table S4. Best-fit parameters from the analysis of K45 $T_{1\varrho}$ magnetization recovery curves, fitted using a sum of three exponential functions according to Equation S1. Reported values are the relaxation times $T_{1\varrho i}$ (ms), the corresponding weight percentages w_i, and the medium relaxation $T_{1\varrho m}$ calculated as the inverse of PWRA.

T (K)	w_1 (%)	w_2 (%)	w_3 (%)	$T_{1\varrho 1}$ (ms)	$T_{1\varrho 2}$ (ms)	$T_{1\varrho 3}$ (ms)	$T_{1\varrho m}$ (ms)
243	7	66	26	0.4	4.2	10.1	2.5
263	22	65	14	0.6	2.6	10.0	1.5
283	48	41	11	0.4	1.3	10.1	0.6
298	49	41	10	0.3	1.1	10.2	0.5
323	68	32	0	0.7	5.0	10.0	1.0
343	18	50	32	0.5	2.7	10.9	1.7

Table S5. Best-fit parameters from the analysis of D45 $T_{1\varrho}$ magnetization recovery curves, fitted using a sum of three exponential functions according to Equation S1. Reported values are the relaxation times $T_{1\varrho i}$ (ms), the corresponding weight percentages w_i, and the medium relaxation $T_{1\varrho m}$ calculated as the inverse of PWRA.

T (K)	w_1 (%)	w_2 (%)	w_3 (%)	$T_{1\varrho 1}$ (ms)	$T_{1\varrho 2}$ (ms)	$T_{1\varrho 3}$ (ms)	$T_{1\varrho m}$ (ms)
243	11	42	48	0.8	4.2	10.8	3.5
263	25	54	20	0.6	2.2	16.0	1.4
283	41	41	18	0.3	0.9	14.5	0.5
298	44	40	16	0.3	1.2	15.2	0.5
323	20	52	27	0.4	2.2	11.1	1.4
343	14	49	37	0.7	4.3	16.1	3.0

S3. Bound Rubber

Table S6. Bound Rubber percentages (BdR, %) obtained by swelling experiments on the indicated samples.

Sample	BdR
REF	23
K15	17
K25	20
K35	18
K45	19
D15	10
D25	11
D35	15
D45	7

S4. DSC experiments

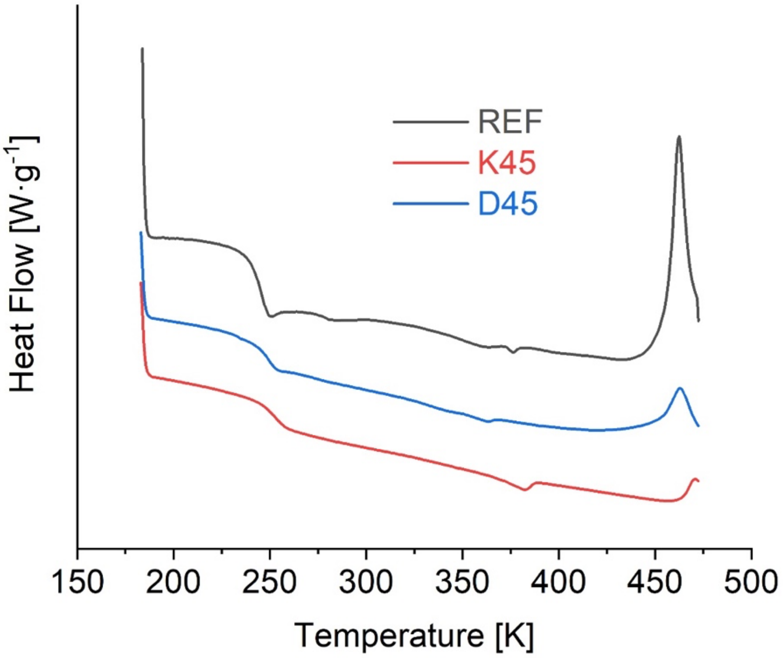

Figure S4. Representative DSC curves for the indicated samples.

S5. MDR experiments

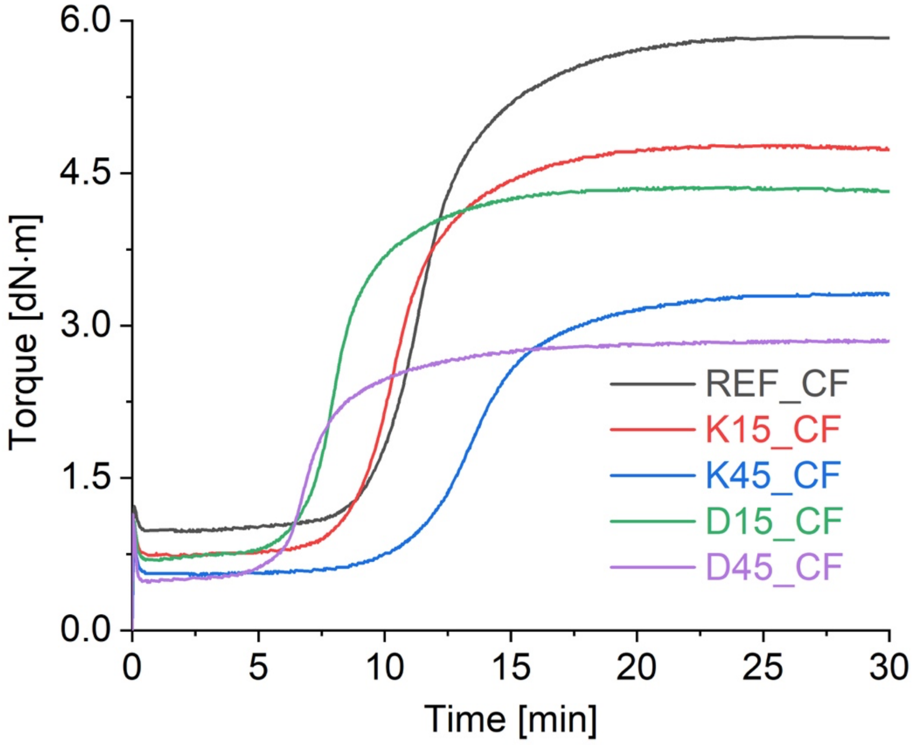

Figure S5. MDR rheograms of the indicated CB-free samples at 443 K.

S6. Stress-strain experiments

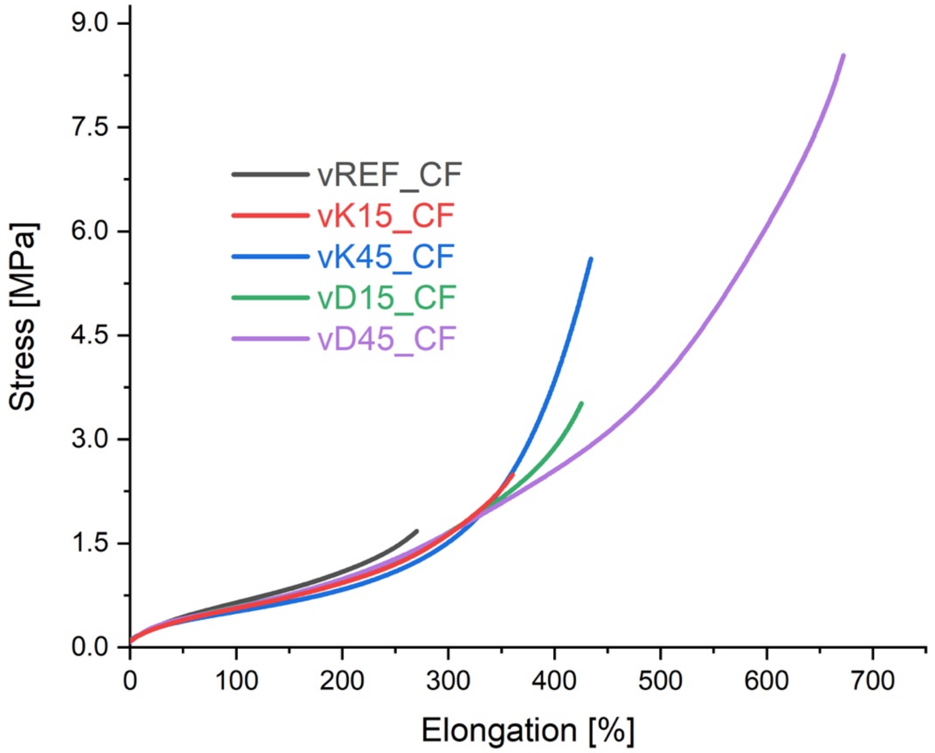

Figure S6. Stress-strain curves of the indicated CB-free samples.
